# Supplementary material for: mRNA localization and thylakoid protein biogenesis in the filamentous heterocyst-forming cyanobacterium Anabaena sp. PCC 7120
Source: J Bacteriol. 2024 Sep 27;206(10):e00328-24. doi: 10.1128/jb.00328-24 (PMC11500504; doi:10.1128/jb.00328-24)
Supplement: Supplemental fgures and tables — Fig. S1 to S7; Tables S1 to S8. [file jb.00328-24-s0001.docx]

**mRNA localization and thylakoid protein biogenesis in the filamentous heterocyst-forming cyanobacterium *Anabaena* sp. PCC 7120**

Kexin Wang, Moontaha Mahbub, Giulia Mastroianni, Ana Valladares and Conrad W. Mullineaux

**SUPPLEMENTARY MATERIAL**

**FIG S1. *cox* mRNA FISH signals in *cox* mutants**

**FIG S2. Effect of nitrogen stepdown on *psaA* mRNA FISH signals in wild type *Anabaena***

**FIG S3. Phylogenetic tree of *Anabaena* and *Synechocystis* RBP sequences**

**FIG S4. PCR segregation tests for *Anabaena* Δ*rbpF*, Δ*rbpG* and Δ*rbpF*/*G* mutants**

**FIG S5. Confocal imaging of chlorophyll fluorescence in *Anabaena* wild type and Δ*rbpG* cells grown diazotrophically**

**FIG S6. Complementation of the *Anabaena* Δ*rbpG* mutation**

**FIG S7. Location of mRNAs in the Δ*rbpF* and Δ*rbpG* mutants**

**TABLE S1**. **Set of 48 FISH probes for *Anabaena psaA* mRNA**

**TABLE S2. Set of 47 FISH probes for *Anabaena psbAI* mRNA**

**TABLE S3. Set of 44 FISH probes for *Anabaena cpcAB* mRNA**

**TABLE S4. Set of 48 FISH probes for *Anabaena cox2* mRNA**

**TABLE S5. Set of 48 FISH probes for *Anabaena cox3* mRNA**

**TABLE S6. *E. coli* strains and plasmids used in this study**

**TABLE S7. Oligonucleotide primers used to generate constructs for deletion of *Anabaena* *rbpG* and *rbpF*.**

**TABLE S8. Oligonucleotide primers used to generate the construct for complementation of *Anabaena* Δ*rbpG*.**


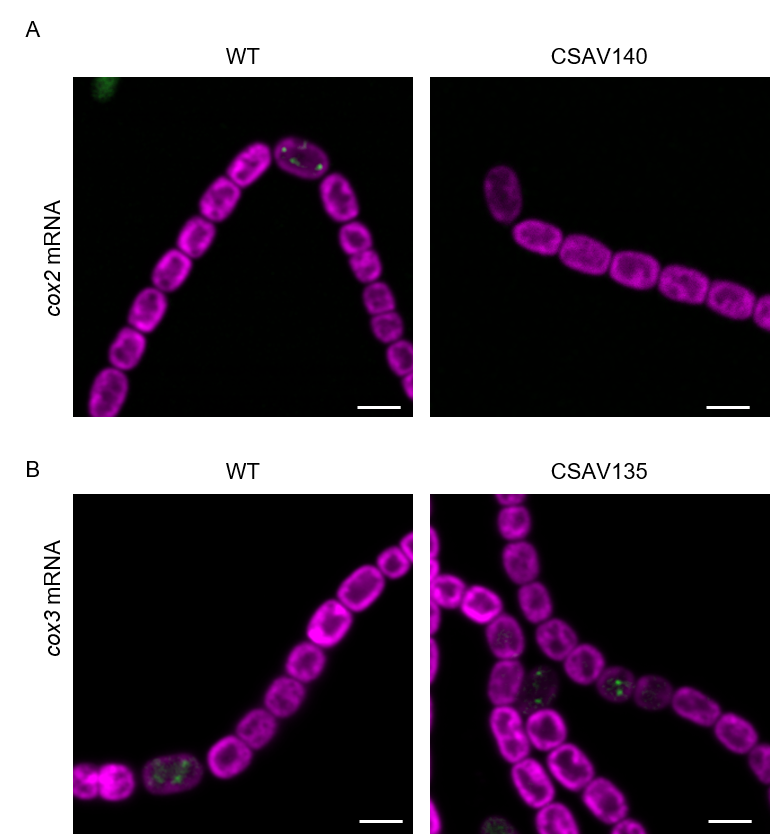


**FIG S1. FISH signals for *cox2* and *cox3* mRNAs in *Anabaena* wild type vs the CSAV140 (*coxB2*::C.S3) and CSAV135 (*coxA3*::C.S3) mutants.** Cells were fixed 12 h after transfer to BG11_0_ medium. Scale-bars: 3 µm.


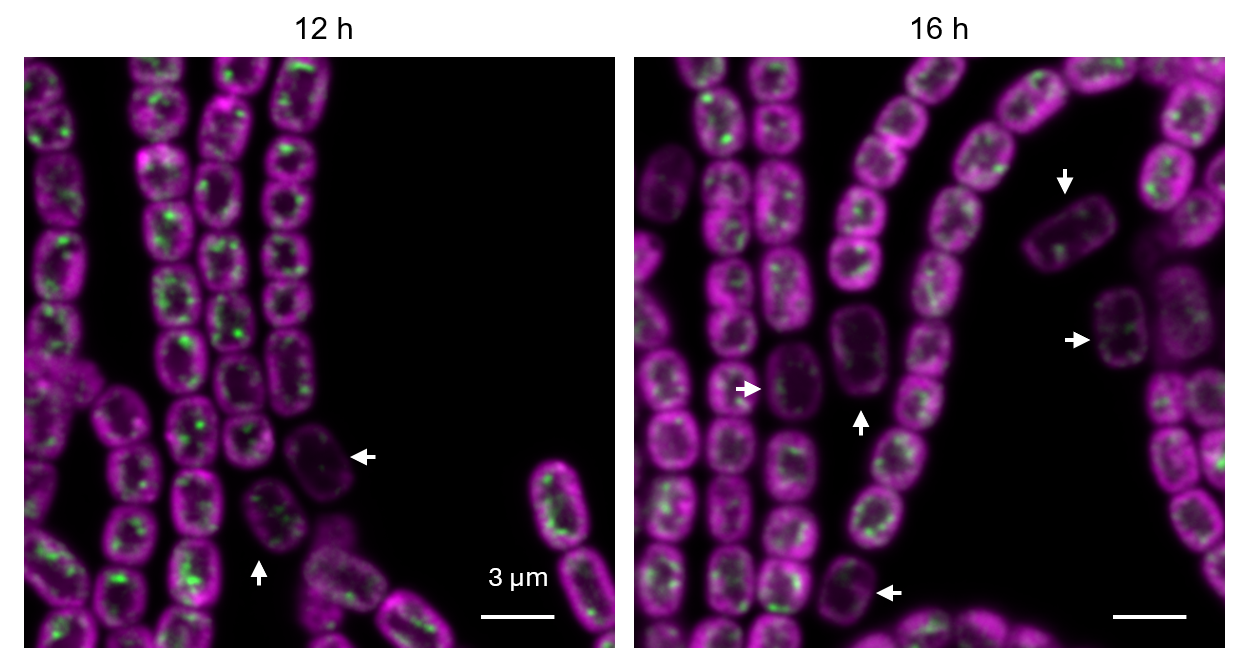


**FIG S2. Effect of nitrogen stepdown on *psaA* mRNA FISH signals in wild type *Anabaena*.** Cells were fixed 12 or 16 h after transfer to BG11_0_ medium. Arrows indicate developing heterocysts. Scale-bars: 3 µm.


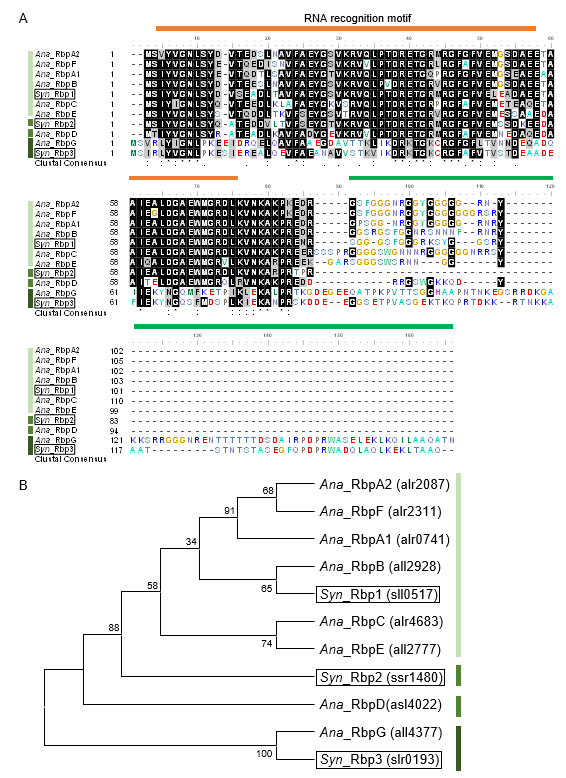


**FIG S3 Multiple alignment and phylogenetic tree of 11 Rbp sequences from *Anabaena* sp. PCC 7120 and *Synechocystis* sp. PCC 6803. A**. Sequences aligned with the ClustalW program on Bioedit. Black background highlights identical conserved residues, and gray background highlights similarity. **B.** Minimum Evolution phylogenetic tree based for the 11 Rbp amino acid sequences. The phylogenetic tree was mapped using the Minimum Evolution method in MEGA X. Bootstrap values based on 500 replicates are shown at nodes. The green bars indicate membership of different classes according to Hamano *et al* (26).


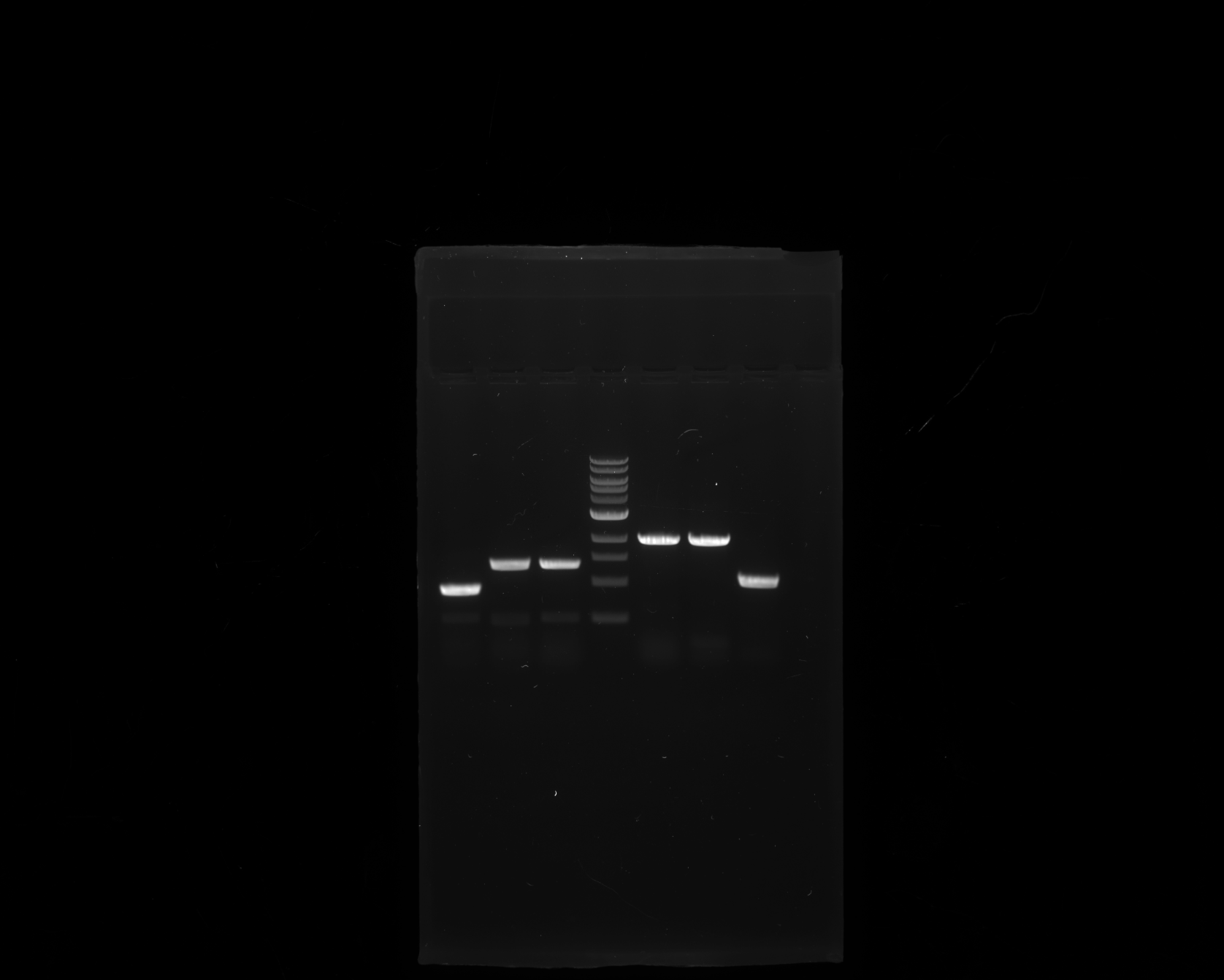


WT

WT

Δ*rbpF/G*

Δ*rbpF/G*

*rbpF::Spec^R^Strep^R^*

*rbpG::Nm^R^*

0.5 kb

1 kb

1.5kb

**2 kb**


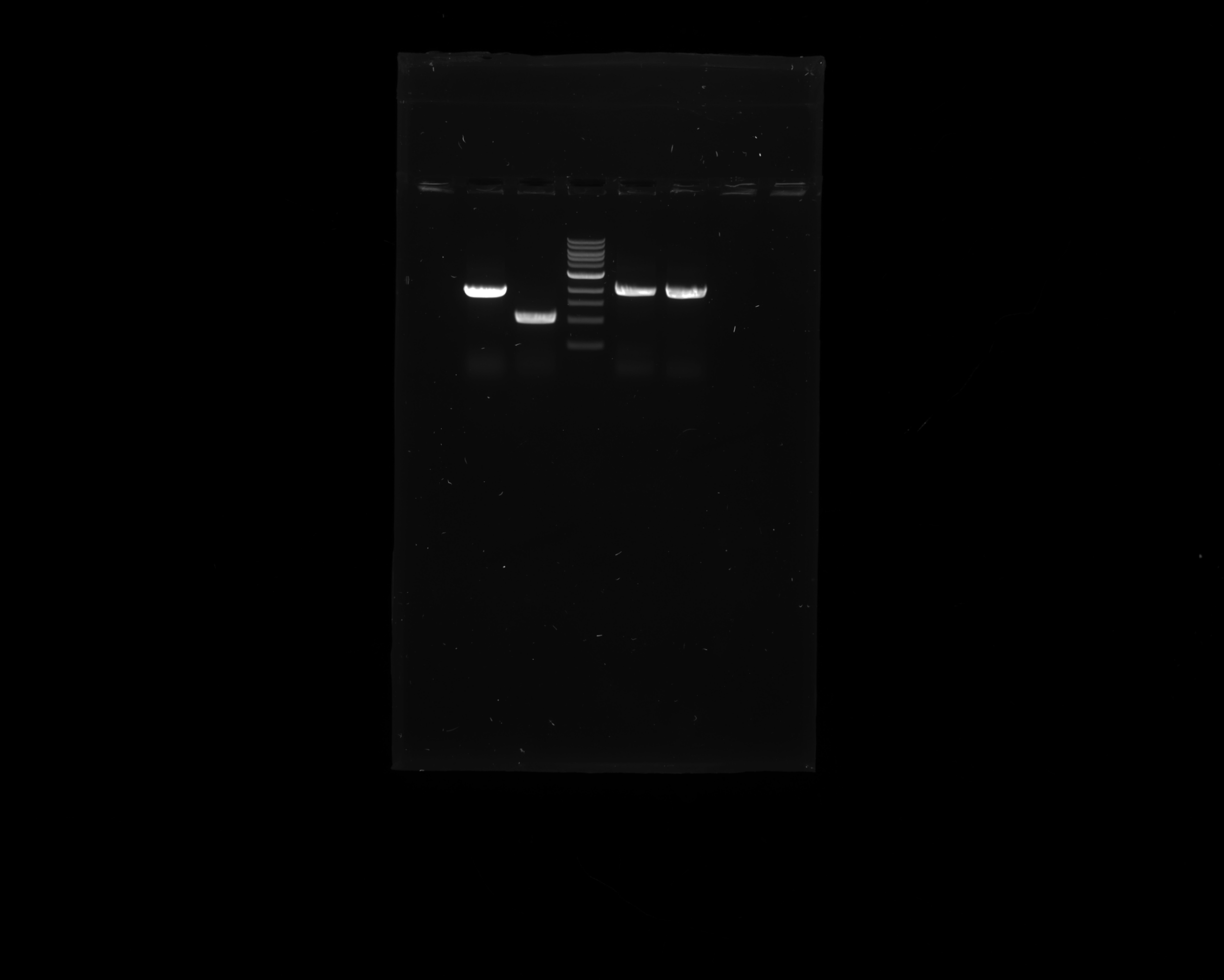


WT

Δ*rbpG*


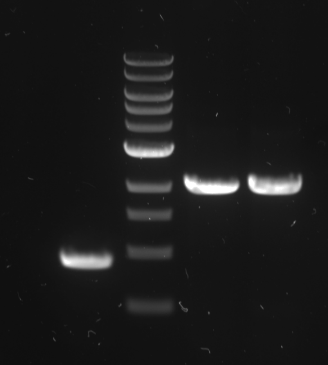


WT

Δ*rbpF*

0.5 kb

1 kb

1.5kb

**2 kb**

*rbpF::Nm^R^*

*rbpG::Nm^R^*

**FIG S4. PCR segregation tests for *Anabaena* Δ*rbpF*, Δ*rbpG* and Δ*rbpF*/*G* mutants**


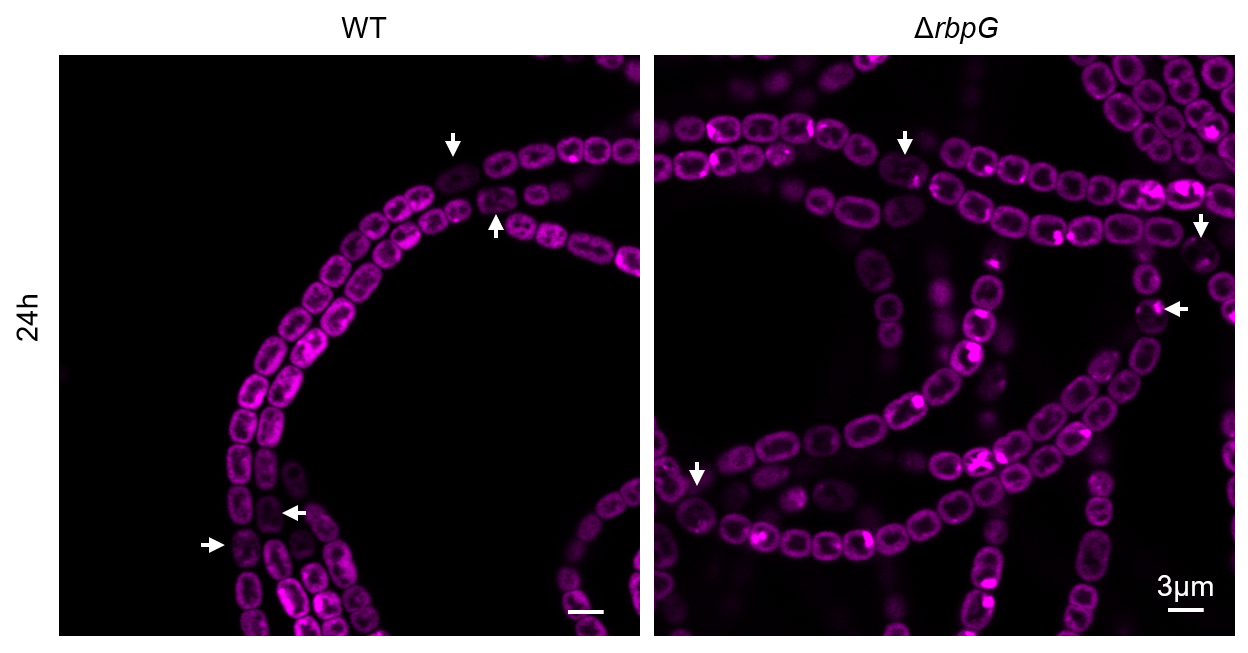


**FIG S5. Confocal imaging of chlorophyll fluorescence in *Anabaena* wild type and Δ*rbpG* cells grown diazotrophically.** Images were recorded 24 h after nitrogen step-down by transfer to BG11_0_ medium. Arrows indicate heterocysts and pro-heterocysts.


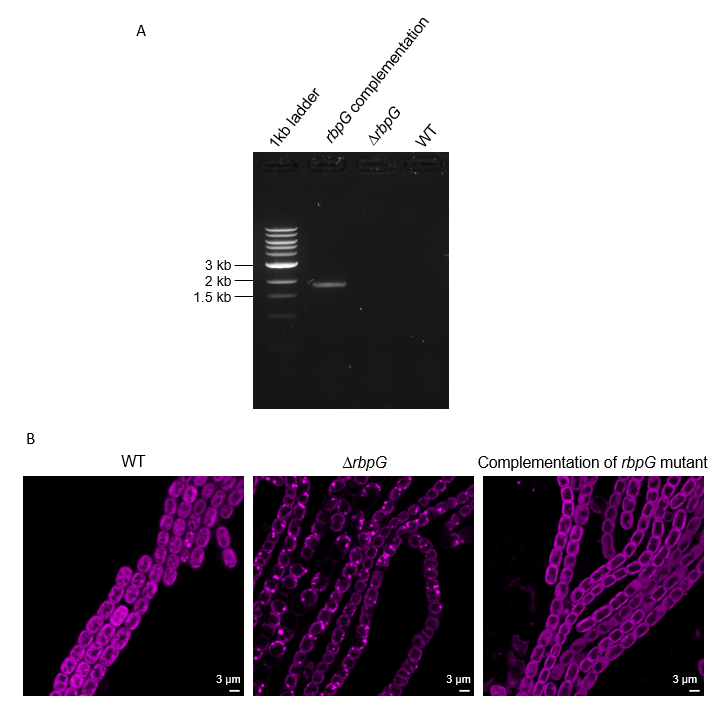


**FIG S6. Complementation of the *Anabaena* Δ*rbpG* mutation.** A. Colony PCR reaction with primers Promoter_RbpG.F and SmR.R (Table S8) to confirm the presence of the plasmid carrying the *rbpG* gene in the complemented strain. B. Confocal imaging of chlorophyll fluorescence in the complemented strain, compared with wild type and Δ*rbpG*. Cells of the 3 strains were grown on BG11 agar plates and imaged in parallel with excitation at 481 nm and emission at 650-720 nm.


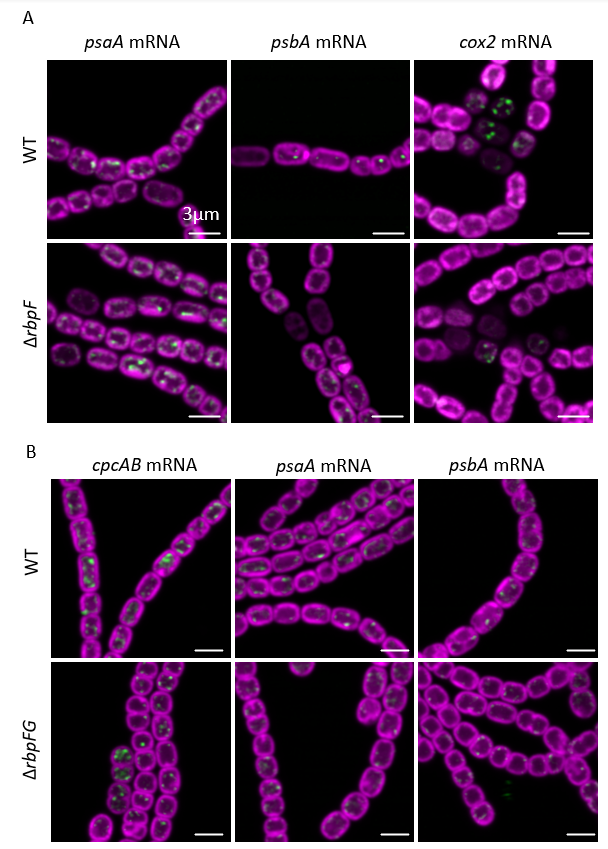


**FIG S7. mRNA FISH signals in the mutants Δ*rbpF* and Δ*rbpF/G* vs wild type *Anabaena*.** TAMRA FISH signals are shown in green and photosynthetic pigment fluorescence in magenta. All scale-bars: 3µm. (**A**) Cells were fixed after 12 h in BG11_0_ medium (for *psaA* and *psbA* mRNAs) and after 16 h in BG11_0_ medium (for *cox2* mRNA) for both wild type and Δ*rbpF*. (**B**) Cells were grown in standard BG11 medium.

**Table S1**. **Set of 48 FISH probes for *Anabaena psaA* mRNA.** Sequences 5’-3’.

| AGGAACCGGATCTTTATCGA | GAGAGTTCTGTCGAAGTGTC |
| --- | --- |
| TATTTTGCGGGAGATGTCTT | ACCAAATTGTCACAACTGCC |
| TTCGCGCCATGGAAAATCAT | CCAGGCTTCGTAGTTAGAAA |
| CAATGGGCCAAACGACTTGA | CCATTCAATATGTCTTGTCC |
| CTGAATACCGTGGAATCCAC | CCGATAGCTGTGCAGTAAAG |
| GAACAAGCCTGCTAATACCA | TTTATGGTAGTGGAACCAGC |
| TCCATTAGCTTGTTGATTGG | TGAGGCAAGGGGATATCTTT |
| AGGCTCTTGTTGAGGATGAA | AGGTGTTAAACCAGCAGCAA |
| AAGAAGTCAGCGTACTGACC | CGGTTACAGGGTTTAAACCG |
| AATGTCAGTCATCCACAAGC | TATTGGTGTCCTGCAATGAT |
| ATTTCTTTGATGCTGTGACC | TAGTTGCTAATTGAGCGTGC |
| ATATGGTGCGCGATGATGAT | TTGCTAAGTATGGGTAGGGA |
| CCAAATGTGGTGGGTGAAGA | CCGCACCATAAATATTGCTG |
| TGCATCTCTGTGACGAATCA | ATACAAACCCAGTTCAGGTG |
| AAACCAAAGCTGTGGAAGCC | GCTGTGTCGGAGAACATATC |
| TTTTGTACCCACTGAGCAAA | CAAAGGCGTAGCTAACAGGT |
| ATAGGCATCATTGCGACTTT | GGTGAATCAAGAAGTCGGCT |
| TGAATGGTGAAGGCGTGGAT | GGCGAACAATACACCTTTCA |
| TCTGGAATCAGACGAGAGCT | GGGAGTTGTACATCCAGAAT |
| CAGACATCAGACTGCATCTT | ATGTGAGACACATTACCTGC |
| ACTTTGGGCAAAGTTACCAC | AAGAAATCACGCAACCAGCC |
| CATAAGCGGACAACGCACTT | ACATTAAGCTGAATGCCCAT |
| ATTATGCGCCCAAACAATGG | TTGAGTAATGCTGAGAGCGC |
| GTAGTGAGCTACACCAACAG | ATGTGTGCGTGGAAGAATGC |

**Table S2**. **Set of 47 FISH probes for *Anabaena psbAI* mRNA.** Sequences 5’-3’.

| TGTTGTAAGGTTGTGGTCAT | CCATACGTTAGCGCTACTAC |
| --- | --- |
| TGATCCATGTGCAGAACCGT | TAAATGCGGTTTTCGGTGCT |
| CAACACGCCGAACCAACCAA | CGGCTAGCAGGGTAGGAATC |
| AGCGATGATGAAGCAGACGG | AATGTCTACTGGAGGTGCAG |
| CTACTGGTTCACGGATACCA | TTTCCGTAGATCAAGGAACC |
| AACTGCACCAGAGATGATGT | CAATAGCGTTGGAGGAAGGA |
| CAGATGGGGTAGAAGTGCAA | CCACTCATCTAAGGAAGCTG |
| ATTACCAATTGGTAAGGGCC | GCATCCGATCAAGAAGTGGA |
| ACTGACGACCGAGGTAGCAA | CGCATACCTAAACGGTAAGA |
| GTAAGCTACGCAGATCCAAG | TAGCAGATGCCAAAGGTGCA |
| GGGTAGATCAAGAATACTGC | AGAGAAGGAACCTTGTCCGA |
| AGATACCCAAGGGCATACCA | ATCATGAAGTTGAAGGTGCC |
| TGTTGTGTTCTGCTTGGAAC | ATGTGGAAGGGGTGCATCAG |
| GAATACACCAGCTACACCCA | TTGCAGAGAACAAGGAACCA |
| GGAAGTTACCAAAGAACCGT | CGGTTGTTTCACGAACCAAG |
| CCGTAGTTCTGAGATTCGAT | TTCTTCTTGACCGAATTTGT |
| CTGCAACGATGTTGTAGGTT | ATCAAACGACCGAAGTAGCC |
| GGCTGTTGTTAAAGGAAGCG | GCTAGGAAGAAGTGCAGTTG |
| GATACCGATTACAGGCCATG | TTACGCCCAACGCGGTAAAC |
| CGTTCAAGTTGAACGCCATT | GTCGATGATGGATTGGTTGA |
| GGTATTGATTACACGACCTT | AGCGCGGTTGATGATGTCAG |
| GCATTACTTCCATACCCAAG | AAGTTGTGAGCGTTACGCTC |
| ACCAGCAGCTAAGTCTAGAG | TTAACGCAACAGGAGCAACT |
| TAACCGTTGATAGCAGGAGC |  |

**Table S3**. **Set of 44 FISH probes for *Anabaena cpcAB* mRNA** (alr0528-0529). Sequences 5’-3’.

| CACCTTGGTAAATACGTCTA | CTCTGGAGTCAGCTTGAGAA |
| --- | --- |
| TGTTCGTTGCTCAGGAACTC | AACATTTGCCAATGCGTCTA |
| AAGCGTTTGTTGCCTTCTTT | CTGGTGATGCGGTTAACAAC |
| TGGTAACGATCGCAGAAGCG | TCAAACAACGCACGAGCAGC |
| AGCAATCAACTGGGGTTGTT | TTGGTGTAAGCGTTACCACC |
| TAAACAAGCAGCCATGCGAC | GCAAGATGATTTCCATGTCG |
| AGGATAGCGTAGGTGACGTA | TAGAACGCTAGCATCGCCTG |
| AAGCCGTTCAAGCAGCGATC | CCAAAGCTTGGTATGTTTCG |
| AGCTACGGAAGAACCAGGAG | CTTTCATTTTTTGAACGCCA |
| GTCGTTAGCGATGCCAACAG | CACCTTTGGTGATTCCGTTG |
| TCAGAGATCAATTGGCTGCA | GCGATCAAAGTAGCTAGCAA |
| TTTAACCAACAGCAGCAGCA | TCGGTAATGGGGGTTTTAAC |
| GGTGTCAGCAGCTGCAATTG | CGGTGTTGCCTAAGAAACGA |
| CATAACGACCACGAGCTGAT | CTTCTAAACTAGCAGCAGCG |
| TTGGAAGTCAAACCACGAGC | ACCATCGATCAAGCGTTGAG |
| TTTGGTAAACAGCTTGGGTT | GTTTGGGTTGTGTAAGGGAA |
| TGTCAGCAGCGAACTGAGGA | ACGAGCGCACTTGGATTTAC |
| GATGATGCGTAGGTAGTGAC | CCACCAGCAACTAAGCTATA |
| TAGGTATTCATCCAAGGGGC | TGATTTCAGCCAAACCAGCA |
| GGAGATAGGTCAAAGGTGCT | TAGAGCTTCAACATACCAGC |
| CATGATTAGCTTTGATGTGC | TTAGCAGCTTGACCACTTAA |
| AGTCGATGTAGGTGTTAGCT | TAGCTGAGAGCGTTGATAGC |

**Table S4**. **Set of 48 FISH probes for *Anabaena cox2* mRNA** (*coxB2* alr2514 - *coxA2* alr2515). Sequences 5’-3’.

| GAGTCCAGAGTGAAACAGGA | TCCAACTACTATTCCAGCAA |
| --- | --- |
| GTTGTGATTTTGACCAATCC | GTTCTGATGCTTGAATAGGT |
| AAATCCGTCTACCAAAGGCG | GCCACAGCAATGGTAAACAT |
| AGAATCGTGCCTTCTACTAC | TACGCCATCGGTATTATCAC |
| GCCGTCCAGAAAATCTCTAA | TAATCCTCCCATTTGGTTAA |
| CTAATGCCGTACCTGAAGAG | GAGGTATCGTTTAGTGTTGC |
| CCCTGGATTAATTGCAGATG | TTGTCGGACTTGCACCAATA |
| GACAACTAAGTCTGCGGTTT | TAACCAAGCAAACTGCATCC |
| CGGAAACACCGTTATCAGGA | AAGTTGTACATCAGCACCTA |
| TCACATCCTGTGCTGAAAGA | ATTGGGGAACCCAGAATGAG |
| AATTGCGTCTTGCTTCAGTC | CTGGTTTGGTAGCTACGAAT |
| CAAAGTTCAGCGCACACTAC | TCCGCATTGAACCATGATAA |
| GGTGTGTGGACAATAACCTG | TGCAAGCCAGCTATCAAACT |
| TTTGTTGTTGAGCAACCTGA | TAGCTGGGTTAACTGCAACA |
| GTGCGAGAAACTCGGATGTT | CTAATTCCCAAATCTTGGGT |
| GCAATGTCTCTAAAGTGGCT | GCGGAATGTGTGGTGGAAAT |
| TTTTTCGGCTGATTGTCATC | AGAGTTAAGCCATGTCCAAC |
| AAAGTAATCTCGCCATTTCC | TGTCACCAGGTATTGGATAC |
| TCAACCCACCGATTAGATAG | TAACTCGGTACGGATAGCGA |
| ATGAAGTCTGCATCAGGTGT | TGCGTTGTACAGATTCGGAT |
| CATGATTGTTCCGTGATTGG | AAAACCCCCAATAGCACTAG |
| CCCAATCATCAAGGGTATGA | CAGTTTGGGAAACGCCATAT |
| GTGGGTTTAACCAAAAGGCG | AGTAACAAGAGTAAGCCGGC |
| AGTCCAACCAGACTGAGAAC | AGTTGGCGCTGTGACTAAAC |

**Table S5**. **Set of 48 FISH probes for *Anabaena cox3* mRNA** (*coxB3* alr2731 – *coxA3* alr2732). Sequences 5’-3’.

| GCCTATTACCAATGTCACAA | ACTGGTAATAGTCACAGCGA |
| --- | --- |
| GTGTAAGCCAGTTTACCAAT | TTAAGATTGATTCAGCCGCC |
| AGATGAATGCACCCATAGTT | CAGAGTGGAGGTAACACCAA |
| GCCCGATGGAATAGTAAAGA | ATCGCTGAGGTCGTTTTCAG |
| ACATTACCTTCAATGTGGGG | GAGGATAGGAATAGCTGTCC |
| AGGTGGCAATCCAAACTACT | GCCCATTTGTTCGTAAATTT |
| CAGGTGTACTAATGCTGTCG | GGTTCAACTAAACCATCCTC |
| GCCCATTGTTTAGCAATTAC | GGTGCTGGTAACATTCTTTT |
| GCGATCGCTTGGTAAATGTA | GAATGCAGCGCCAATTTTAC |
| ATAGAAGCCGTGGAGTACAT | GGATGATGTCTTGCTTGAGG |
| TCAAAGTCGATGTTGTGGTT | TATTTACCTTCACGGATGGG |
| CCACTATATTGAGAGTCGGT | CTACATTGGCTTGCATAGTG |
| ATACTCTTCAGGAGATTCCA | GCTATTTTGGCTAACCATTT |
| ATGCTTGATTGTAGGCAGTG | ATGCTTTGTGCATATTCAGC |
| CAACCAGTTTTGACTTGCTG | AATTAACCAAAGGTGCGGCG |
| ATGTTGGTCATTTAACCAGG | GAGTTGAACGCCTTCTATGG |
| AGCTGAAATATTCCTTCCAG | CCAATAACCTTGTGGTCATG |
| GGAGGTTACAAGGTATTGGA | GTATCATGGCGAAGATACCG |
| TGATTCGGGGGTGATGAGTT | ATAAACGGTGCGGTCGATGA |
| GTCCACAAGAACAGCATCAC | TAGCTAGTCCAACGAGTGAG |
| CCCAATCATCAAGGGTACTA | CAAGAGAATCCCGACTACGG |
| CCAGGGACAAAGAAGCTAGT | GTACTTACCGGAGGATAAGC |
| TAAGTTACCTGTGGGATTCT | ATAGCAACTGCTAATAGCCA |
| CCATAATTGAGGAGACACCG | GTGGTTACAAAGTTCACAGC |

**Table S6** ***E. coli* strains and plasmids used in this study**

| **Strain or plasmid** | **Resistance** | **Source or reference (s)** |
| --- | --- | --- |
| NEB® 5-alpha (#C2987) | - | New England BioLabs |
| HB101 (+pRL623) | Cm^R^ | Dr. Dennis Nürnberg |
| ED8654 (+pRL443) | Amp^R^+Tc^R^ | Dr. Dennis Nürnberg |
| pRL271B | Cm^R^ | Modified by Dr. Fabian Conradi |
| pRL623 | Cm^R^ | Elhai *et al.*, 1997 (39) |
| pRL443 | Amp^R^+Tc^R^ | Elhai *et al.*, 1997 (39) |
| pAM4688 | Nm^R^ | Chen *et al.*, 2016 (41) |

**Table S7: Oligonucleotide primers used to generate constructs for deletion of *Anabaena* *rbpG* and *rbpF*.** Lower-case letters indicate overhangs for Gibson assembly.

| Primer name | Sequence (5’ – 3’) |
| --- | --- |
| **Δ*rbpG* (all4377)** |  |
| US-S-all4377-PstI | gcgaaagcttgcatgcctgcagCATCCACACTGATCAACAA |
| US-A-all4377 | gccgggtaatactcgtcaacGAACTATACCTCCAGAGTTAAA |
| KmR-S | GTTGACGAGTATTACCCGGCATT |
| KmR-A | TTAGAAAAACTCATCGAGCATCAAA |
| DS-S-alr4377 | tgctcgatgagtttttctaaGTGAGCAGACTGAGAAATTAAA |
| DS-A-all4377-BamHI | gcgatctgtctatttcgtggatccCTCTCTTCTTTTGCGAATCA |
| Seq-S-all4377 | AGGGCAGACAAAAAATTATT |
| Seq-A-all4377 | AAGATAGAAGAAGGTTTAGT |
| **Δ*rbpF* (alr2311)** |  |
| US-S-alr2311-PstI | gaaagcttgcatgcctgcagGTGACCACAGTTCACCTTTA |
| US-A-alr2311-KmR | gccgggtaatactcgtcaacTCTTTTCTCTCCAAAATCATGG |
| US-A-alr2311-SmR | gcgatcaccgcttccctcatTCTTTTCTCTCCAAAATCATGG |
| DS-S-alr2311-SmR | tcaccaaggtagtcggcaaataaCTAAATTCTTGAGGTCAGAC |
| DS-S-alr2311-KmR  DS-A-alr2311-BamHI | tgctcgatgagtttttctaaCTAAATTCTTGAGGTCAGAC  gcgatctgtctatttcgtggatccTCTCGAACGTAGGATTGATA |
| Seq-S-alr2311 | AAGCGGGAATTTATGACTGT |
| Seq-A-alr2311 | CTGGTGCTATCGGTTGAGTG |
| SmR-S | ATGAGGGAAGCGGTGATCGC |
| SmR-A | TTATTTGCCGACTACCTTGGTGATC |

**Table S8: Oligonucleotide primers used to generate the construct for complementation of *Anabaena* Δ*rbpG*.** Lower-case letters indicate overhangs for Gibson assembly.

| **Primer name** | **Sequence (5’ – 3’)** |
| --- | --- |
| Promoter_RbpG.F | aaggagcccttcaccCACAACTAGCGAGCTTGGTTG |
| Promoter_RbpG.R | atcaccaaggtagtcggcaaataaTTAATTTGTGGCTTGAGCTGCTAG |
| SmR.F | tagcagctcaagccacaaattaaTTATTTGCCGACTACCTTGGTGATC |
| SmR.R | AGTTTGTACAAGAAAGCTGGGTatgagggaagcggtgatcg |
| Vector.F | caccgcttccctcatACCCAGCTTTCTTGTACAAACTC |
| Vector.R | agctcgctagttgtgGGTGAAGGGCTCCTTCTTAAAG |
